# A novel Strategy of Lock-in Effect between Conjugated Polymer and TiO_2_ towards Dramatic Enhancement of Photocatalytic Activity under Visible Light

**DOI:** 10.1038/s41598-020-63623-2

**Published:** 2020-04-16

**Authors:** Linlin Liu, Wei Jiang, Xingyue Song, Qian Duan, Enwei Zhu

**Affiliations:** 1 School of Materials Science and Engineering, Changchun University of Science and Technology, Changchun, 130022 P. R. China; 2 Key Laboratory of Functional Materials Physics and Chemistry of the Ministry of Education, Jilin Normal University, Changchun, 130103 P. R. China; 3 Key Laboratory of Preparation and Application of Environmental Friendly Materials, Jilin Normal University, Ministry of Education, Changchun, 130103 P. R. China; 4 School of Environmental Science and Engineering, Jilin Normal University, Siping, 136000 P. R. China

**Keywords:** Chemistry, Materials science

## Abstract

To design novel conjugated polymers and improve interfacial interaction with semiconductor is one of directions to develop high-efficient photocatalysts with harvesting photons and boosting catalytic activities. Herein, two novel linear conjugated polymers poly[(thiophene-ethylene-thiophene)-thiophene] (PTET-T) and poly[(thiophene-ethylene-thiophene)-thiophene-3-carboxylic acid] (PTET-T-COOH) were successfully synthesized by a simple Stille coupling reaction. Their heterojunction with TiO_2_, i.e, PTET-T/TiO_2_ (C1) and PTET-T-COOH/TiO_2_ (C2), exhibited outstanding photocatalytic activity for degrading Rhodamine B, methylene blue and tetracycline. The energetic “lock-in effect” between PTET-T-COOH and TiO_2_ through carboxyl groups and hydroxyl groups interaction has been proved to greatly improve the interface charge transfer ability and suppress the electron-hole recombination in PTET-T-COOH/TiO_2_. Thus, by regulating the dosage of polymers, the 15% PTET-T-COOH/TiO_2_ showed the optimized photocatalytic activity with excellent chemical stability, and its kinetic rate constant was determined to be 41.7 times of that of TiO_2_. This work provided a new effective strategy of designed and explored organic semiconductor-inorganic heterojunction photocatalysts with broaden absorption, repeatability and high-charge mobility.

## Introduction

Nowadays, severe water contamination caused by the release of dyes, antibiotics, drugs, and phenolic pollutants become an urgent issue to be addressed^1–6^. Semiconductor photocatalysis is one of promising techniques to degrade these water contaminations^7–10^. So far, the wide-bandgap metal-semiconductors such as TiO_2_, ZrO_2_, ZnO, and SrTiO_3_ as popular photocatalysis materials have been widely used^11–14^. As one of the most promising catalysts, TiO_2_ has demonstrated successful performances in degrading organic pollutants with the merits of excellent chemical stability, low-cost, nontoxic and redox capability^15^. However, the poor carrier separation efficiency and invisible-light responsiveness are the huge obstacles for their practical application^16,17^. The TiO_2_/semiconductors heterojunction photocatalysts have been proved to be a valid mean of optimizing the photocatalytic activity via improving visible-light responsive ability and the efficiency of photo-generated electron-hole separation^18–22^. Unfortunately, the mismatched energy level, full visible-light-responsive range and high charge separation efficiency are still challenging to tune and control for achieving high-efficient photocatalysts.

Most recently, conjugated polymers as the useful organic semiconductors, due to their ability of light absorption and well-tuned energy levels, attract a mass of attention for their promising applications in visible-light responsive pollutant degradation. The conjugated polymers/TiO_2_ heterojunction might be a promising strategy to adresss above issues. In this heterojunction, the conjugated polymers as p-type donor materials can serve as one of the best photosensitizers^23,24^. Polythiophene (PTh) and its derivatives, have been widely explored in the field of environmental governance thanks to their easy to synthesize, long-time stability, high carrier migration efficiency, and controllable narrow band gap. The polythiophene/TiO_2_ composites had attracted widespread attention for degradation of various dye^25–28^, which have been shown to effectively lower the band gap by allowing greater adsorption in the visible region and prevent the recombination of photoelectrons and holes. PTh can be coated on the surface of metal oxides, act easily as a sensitizer to metal oxides^29^. However, the poor stability for long term application was not mentioned in above experiments, or the decolorization rate of organic pollutants was only about 60% after three repetitions. Meanwhile, the wavelength of the conjugated polymer optical response used in the above study is almost below 600 nm, which cannot be effectively utilized in the visible band. Last but not least, the bonding way of polythiophene and TiO_2_ heterojunction determines the photogenerated charge separation and transfer capacity. Thus, a wise strategy is necessary to be proposed to construct a novel polymer/TiO_2_ composites with the better stability and more effective photocatalytic ability for pollutant removal^30^.

The “lock-in effect” has been used for molecular conformation in organic semiconductors. It facilitates the intramolecular delocalization of π-electrons along the backbone, thus enhancing intramolecular charge transfer and reducing the band gap^31^. In our paper, we firstly use “lock-in effect” to open concepts to bridge gaps between the polymer and TiO_2_ composites.

Herein, we designed and synthesized two novel thiophene-based conjugated polymers poly[(thiophene-ethylene-thiophene)-thiophene] (PTET-T) and poly[(thiophene-ethylene-thiophene)-thiophene-3-carboxylic acid] (PTET-T-COOH) with side-chain carboxyl groups. The ethylene in PTET-T could effectively extend π-conjugated system so as to expand visible-light-responsive range, and the carboxyl in PTET-T-COOH could boost the photocatalytic ativity due to the “lock-in effect” between PTET-T-COOH and TiO_2_ through carboxyl groups and hydroxyl groups interaction. The heterojunction PTET-T/TiO_2_ (C1) and PTET-T-COOH/TiO_2_ (C2) were characterized by SEM, TEM, XRD, XPS, and DRS. The possible mechanism of the superior photocatalytic activity was discussed in detail based on photocurrent, electrochemical impedance spectroscopy (EIS), electron spin resonance (ESR) tests, gas chromatography and mass spectrometry (GC-MS) as follow.

## Results

### Morphologies and structure analyses

**Figure 1:**
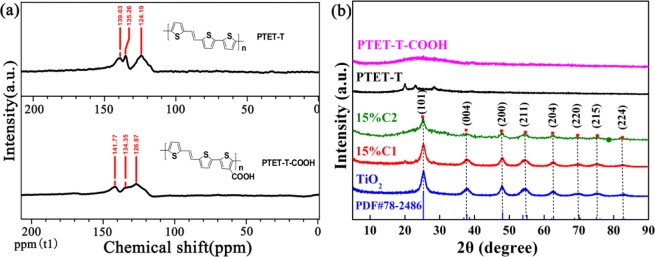
(**a**) Solid-state ^13^C NMR of PTET-T and PTET-T-COOH, (**b**) XRD patterns of TiO_2_, PTET-T, PTET-T-COOH, 15% C1, and 15% C2. (This image is created by SciDAVis, which is a free application for Scientific Data Analysis and Visualization. http://scidavis.sourceforge.net/).

Solid-state ^13^C NMR spectra were employed to confirm the chemical structure of the conjugated polymer PTET-T and PTET-T-COOH, which were prepared with a Pd(0)-catalyzed Stille coupling reaction according to literature^32^. Broad peaks at 139.03, 135.26, and 124.19 ppm were observed in the Fig. 1a, corresponding to aromatic carbon signals of polymer PTET-T. Besides, the aromatic carbon signals of PTET-T-COOH were located at 141.77, 134.35, and 126.87 ppm, which were attributed to electron-withdrawing groups of PTET-T-COOH. Importantly, it was certified that the conjugated polymers had been successfully synthesized and aromatic signals agreed well with the proposed structures of PTET-T and PTET-T-COOH.

As shown in Fig. 1b, TiO_2_ exhibited the XRD pattern of anatase TiO_2_ with characteristic diffraction peaks at 2θ = 25.3°, 37.7°, 47.9°, 54.4°, and 62.6° according to JCPDS card No. 78–2486. Notably, there were no peaks observed in PTET-T and PTET-T-COOH due to their amorphous networks^33^. Compared to pure TiO_2_, the 15% C1 and 15% C2 showed the same XRD pattern, while the intensity of the (101) crystal plane of 15% C2 weaken slightly without significant changes in the peak position due to introduction of PTET-T-COOH. It was indicated that the heterojunction structure had been prepared successfully without impurities generation.

**Figure 2:**
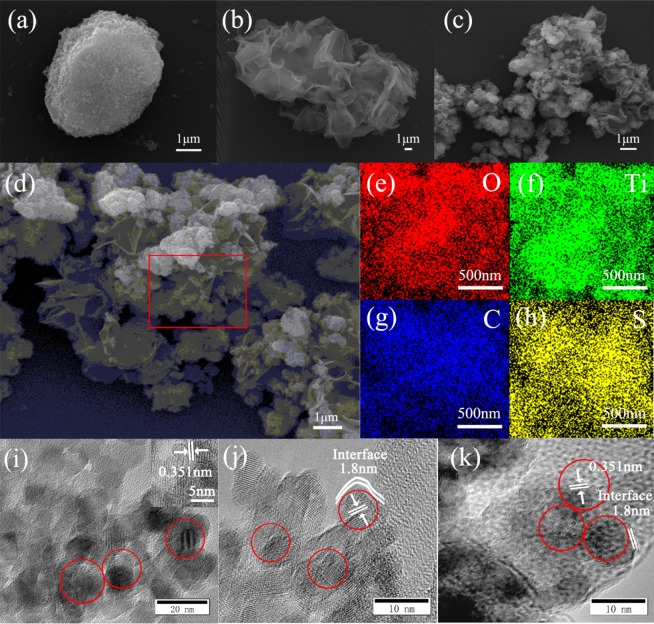
SEM images of (**a**) TiO_2_, (**b**) PTET-T, (**c**) 15% C1, (**d**) 15% C2. The element mappings of (**e**) O, (**f**) Ti, (**g**) C, (**h**) S. And TEM images of (**i**) TiO_2_, (**j**) 15% C1, (**k**) 15% C2. (This image is created by WPS Office. https://www.kdocs.cn/welcome#home).

The surface morphology and the element mappings of Ti, O, S, C areas of heterojunction were given in Fig. 2(a–h). The micromorphology images of TiO_2_, 15% C1, and 15% C2 were further shown in Fig. 2(i–k). The polymers showed film-like morphology among the TiO_2_ particle with a diameter of about 1μm, which can be seen from Fig. 2(a–d). The element mapping images of 15% C1 and 15% C2 (Fig. 2e–h) confirmed that elements including O, C, S, and Ti existed in the as-prepared heterojunction. As presented in Fig. 2i, the diameter of TiO_2_ nanopartikel was about 10 nm, the 0.351 nm lattice structure of anatase TiO_2_ was observed clearly, which was consistent with the results of XRD patterns. There was no visible agglomeration observed and the interface thickness between the crystallized TiO_2_ and amorphous polymer (the disorderly flocculent structure) was clearly found about 1.8 nm in Fig. 2(j,k). According to previous investigation^27^, the optimal dispersion and well interface thickness of the photocatalysts played the significant role in the photocatalytic reaction.

**Figure 3:**
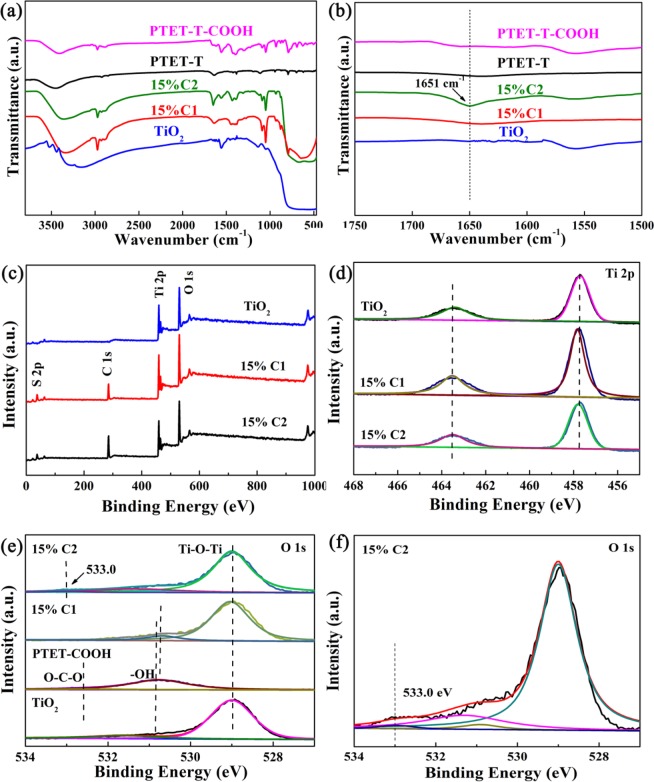
(**a**) FT-IR spectra and (**b**) The enlarge spectra of as-prepared samples. XPS spectra of as-prepared samples (**c**) Survey spectra, (**d**) Ti 2p, (**e**) O 1 s. (**f**) High resolution O 1 s XPS spectra of 15% C2. (This image is created by SciDAVis, which is a free application for Scientific Data Analysis and Visualization. http://scidavis.sourceforge.net/).

FT-IR spectra as depicted in Fig. 3a, TiO_2_ showed a strong and broad absorption band at 550–500 cm^−1^ and 3000–3500 cm^−1^, corresponding to the characteristic absorption peaks of Ti-O-Ti bond and O-H groups, respectively^27^. The absorption peaks at 1387, 1046 and 617 cm^−1^ were attributed to C=C symmetric stretching vibration, C-H in-plane deformation and C-S-C ring deformation stretching of thiophene ring in PTET-T and PTET-T-COOH, respectively. The carboxyl O-H of PTET-T-COOH was observed at 3064 cm^−1^. The bands at 3500 and 3110 cm^−1^ was assigned to the O-H stretching of water in KBr^34^. The FT-IR spectra of 15% C1 and 15% C2 confirmed that the heterojunction composed of PTET-T or PTET-T-COOH and TiO_2_ were successfully prepared. Surprisingly, a new absorption peak emerged at 1651 cm^−1^ in the 15% C2 composite (Fig. 3b), indicating that interfacial interaction between the carboxyl groups of PTET-T-COOH with the TiO_2_ surface over the reaction time could be formed^35^.

The survey XPS spectra of TiO_2_, 15% C1 and 15% C2 in Fig. 3c showed the chemical compositions of Ti 2p, S 2p, O 1s, and C 1s elements in agreement with the results of XRD and the element mapping. Both of TiO_2_ and the heterojunction showed the binding energy at 457.6 and 463.5 eV assigning to Ti 2p_3/2_ and Ti 2p_1/2_ without noticeable shift (Fig. 3d). The high-resolution O 1s XPS spectra could be illustrated into four characteristic peaks at 529.0, 531.6, 532.6, and 530.7 eV, which were assigned to Ti-O-Ti bond and O-H on the surface of TiO_2_ and O-C-O/O-H from PTET-T-COOH (Fig. 3e). Compared to 15% C1, the new peak at 533.0 eV was taken for interfacial interaction in the O1s spectrum of 15% C2 (Fig. 3f) after the heterojunction forms, confirming that PTET-T-COOH was bound onto TiO_2_ by the “lock-in effect” via carboxyl groups and hydroxyl groups interaction on the surface of TiO_2_^27^. According to the results of FT-IR and XPS spectra, the “lock-in effect” would facilitate the intermolecular interaction of heterojunction, thus enhancing the electron injection and interfacial photogenerated carrier separation, which was critical to the visible-light photocatalytic competence of the heterojunction.

**Figure 4:**
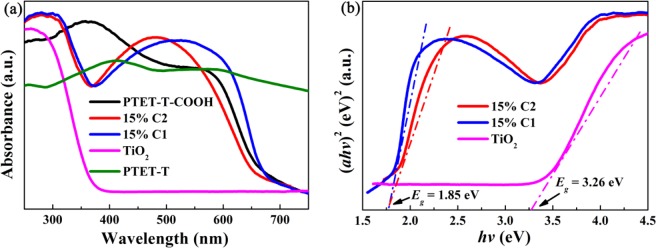
(**a**) UV-Vis DRS of TiO_2_, PTET-T, PTET-T-COOH, 15% C1, and 15% C2. (**b**) Plots of the (ahυ)^1/2^ versus hυ for TiO_2_, 15% C1, and 15% C2. (This image is created by SciDAVis, which is a free application for Scientific Data Analysis and Visualization. http://scidavis.sourceforge.net/).

As presented in Fig. 4a, TiO_2_ showed no visible-light absorption ability due to its wide-bandgap, while the pure polymers displayed a typical absorption band in UV and visble range and an absorption edge at approximately 650 nm^36,37^. For the 15%C1 and 15% C2 samples, the optical absorption region had a marked widening, which gived the credit to the the polymer. The obvious absorption edge of 15% C1 and 15% C2 extended to around 650 nm. Acorrding to the formula of the band gap width: *ahυ* = a (*hυ*-E_g_) ^n/2^, where, a, E_g_, α, *hυ* represent the light frequency, the band gap energy, the absorption coefficient and the Plank constant, respectively. It can be concluded in Fig. 4b that the E_g_ of TiO_2_ was 3.26 eV, the E_g_ of 15% C1 and 15% C2 was about 1.85 eV. Obviously, the conjugated polymers taken on the visible-light sensitizer were of vital importance for prolonging the response spectrum of composites and can improve the utilization of solar energy.

### Photocatalytic degradation performance

**Figure 5:**
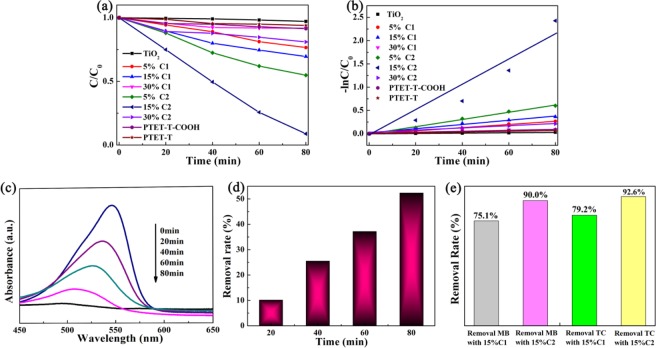
Photodegradation of RhB under visible-light irradiation in the presence of PTET-T, PTET-T-COOH, TiO_2_, C1, and C2 composites.(**a**) Photocatalytic degradation efficiencie between C/C_0_ and irradiation time. (**b**) Reaction kinetics (ln (C_0_/C) = −K_app_t). (**c**) The degradation rate and (**d**) TOC removal efficiency for RhB with the 15%C2. (**e**) The degradation rate of MB, TC with 15% C1 and 15% C2. (This image is created by SciDAVis, which is a free application for Scientific Data Analysis and Visualization. http://scidavis.sourceforge.net/).

The photocatalytic abilities of different catalysts were conducted through photocatalytic degradation of RhB, MB, and TC as target contaminants under visible-light. As displayed in Fig. 5a, TiO_2_ showed no obvious photoactivity because of the limitation wide band gap under visible light. It was found that the degradation ratio of 10 ppm RhB for PTET-T and PTET-T-COOH with broad absorption ability were only 7% and 9% after 80 min of visible light irradiation. In contrast, the degradation rate of RhB significant increased since the polymer added from 5 wt% to 15 wt%. The photocatalytic abilities were promoted gradually with the content of the polymer increased. The maxima was 91.2% within 80 min when the content of PTET-T-COOH is 15 wt%, which is about three times than that of 15% C1. However, the degradation rate was reduced with excessive increasing the content of conjugated polymer, which might hinder the effective charge transfer for redox reactions at the heterojunction interface and decrease in the catalytically active surface area to reduce the efficiency of the charge transfer for redox reactions^38^. The heterostructure C2 had an obvious advantage over C1 in photocatalytic performance, which was due to the interface interaction generated by the “lock-in effect”, which speeds up the separation efficiency between photogenic carriers. All the degradation progresses were presented to accord with first-order dynamic models (Fig. 5b) and the kinetic rate constant of 15% C2 was 41.7 times of that of TiO_2_. Moreover, Fig. 5c displayed the curves of the absorption change of RhB with 15% C2, in which the absorption peaks of RhB gradually disappear during the degradation process. Figure 5d showed the TOC results of RhB over 15% C2 catalyst. Clearly, the TOC removal rates gradually increased within 80 min. The max mineralization rate of 15% C2 was 52.34%. It was surprise to find that the 15% C1 and 15% C2 also showed effective photocatalytic activity during the photodegradation of MB and TC (Fig. 5e).

**Figure 6:**
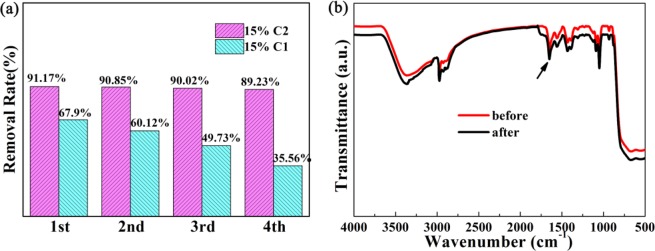
(**a**) Four reaction cycles of RhB with 15% C1 and 15% C2. (**b**) FT-IR spectra of 15% C2 before and after four times photocatalytic degradation of RhB. (This image is created by SciDAVis, which is a free application for Scientific Data Analysis and Visualization. http://scidavis.sourceforge.net/).

The photocatalytic stability was evaluated through cycling measurement in photocatalytic degradation of RhB. As showed in Fig. 6a, maintaining nearly 35% of the photocatalytic activity for 15% C1 was observed after four recycles under the visible light irradiation. However, the photocatalytic performance for 15% C2 maintained about 90% and showed excellent photocatalytic stability under the same condition. The latter presented the stability of their interaction before and after photocatalytic degradation of RhB after four cycles. In order to further confirm the reusability of photocatalyst, 15% C2 sample after recycles was evaluated again through FT-IR absorption spectra (Fig. 6b), SEM, TEM, and XPS spectra (Figure S2). Obviously, the microstructure and chemical compositions had not been changed, which confirmed that as-prepared sample 15% C2 possessed the high chemical stability. The above experimental results indicated that the “lock-in effect” of heterostructure was of great benefit to the stability and reusability of 15% C2 composites.

### Photocatalytic mechanism discussion on heterojunction

**Figure 7:**
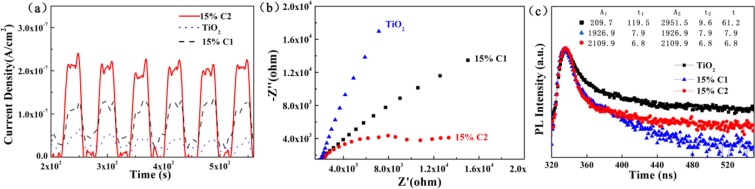
(**a**) Photocurrent intensity, (**b**) EIS spectra comparison, (**c**) TR-PL spectra of TiO_2_, 15% C1, and 15% C2. (This image is created by SciDAVis, which is a free application for Scientific Data Analysis and Visualization. http://scidavis.sourceforge.net/).

It was found that the photocurrent response revealed a relatively stable trend with each light-off and on, in which the 15% C2 showed more powerful photocurrent density than that of the others in the Fig. 7a. The above result illustrated that 15% C2 had the highest migration efficiency of electron-hole pairs^39^. Moreover, photocurrent intensity of 15% C2 was almost 2.5 times higher than that of 15% C1 due to the “lock-in effect” between the PTET-T-COOH and TiO_2_. Meanwhile, this result was evidenced by EIS analyses of TiO_2_, 15% C1, and 15% C2, respectively. The smallest interfacial resistance of 15% C2 was observed compared that of TiO_2_ and 15% C1 (Fig. 7b). In a word, 15% C2 signified to hampered significantly the recombination of electron-hole pairs and the quicker interface charge diversion ability by the strategy of “lock-in effect” of heterostructure^40^. Meanwhile, in Fig. 7c the TR-PL lifetime τ_av_ of the TiO_2_, 15% C1 and 15% C2 were 61.20 ns, 7.89 ns and 6.80 ns, respectively. The achieved result suggested that the strong interfacial interaction of 15% C2 between PTET-T-COOH to TiO_2_ provided an additional deactivation pathway for the transferred electron leading to a remarkable decrease in the TR-PL lifetime^41–44^. Notably, the “lock-in effect” of 15% C2 between PTET-T-COOH and TiO_2_ played a vital role in the recombination of e^−^ / h^+^ and improving the transferability of photogenerated carriers and ultimately contributing to photocatalytic activity^45,46^.

**Figure 8:**
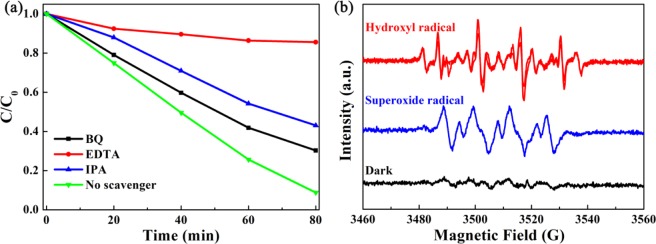
(**a**) Trapping measurement of 15% C2 with different capture reagents (BQ → ·O_2_^-^; EDTA → h^ +^ ; IPA → ·OH) for photodegradation of RhB. (**b**) ESR spectra of DMPO-·OH and DMPO-·O_2_^-^ for 15% C2. (This image is created by SciDAVis, which is a free application for Scientific Data Analysis and Visualization. http://scidavis.sourceforge.net/).

A trapping experiment was used to explore the main active species generated in the degradation system. In Fig. 8a, the photocatalytic activity of RhB exhibited aspecific inhibitory effect with the varying scavenger. It can be seen the addition of EDTA as scvenger of h^+^ had a significant impact on the test system, which the degradation rate of RhB was 15%. In addition, the active species ·OH and ·O_2_^*−*^ (IPA and BQ as the capture reagents) were also present during degradation. The degradation rate of RhB decreased from 91.2% to 57% and 69.8%. In summary, the active species of h^+^, ·OH and ·O_2_^*−*^ produced in the degradation process were successfully testified and played essentials roles in the degradation of RhB. ·OH and ·O_2_^*−*^ active species were also evidenced by ESR measurement. In Fig. 8b displayed that 15% C2 heterostructure in water produced visible four groups of signals for several cycles of intermittent on-off irradiation, demonstrating that ·OH active species were produced in the system. Likewise, four active groups of signals were also scanned in the DMPO-·O_2_^*−*^ ESR spectra under the same condition, which meant that·O_2_^*−*^ active species was also effected. The result was consistent with the active species of trapping experiment.

**Figure 9:**
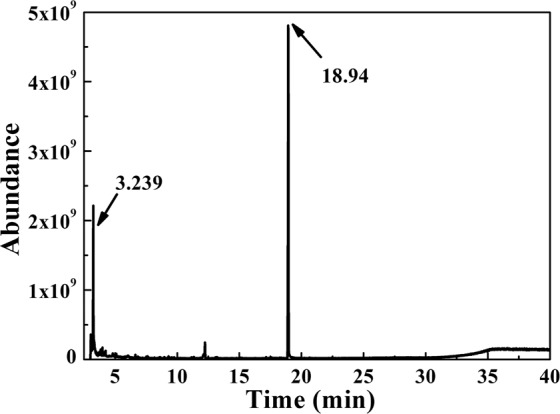
Gas chromatographs of RhB degradation residues over 15% C2. (This image is created by SciDAVis, which is a free application for Scientific Data Analysis and Visualization. http://scidavis.sourceforge.net/).

According to previous publications^47,48^, the possible photocatalytic degradation of RhB included four main steps: N-deethylation, chromophore cleavage, opening ring and mineralization^49^. The dominating peaks in gas chromatographs of RhB degradation residues were at the retention time of 18.94 min and 3.239 min, respectively(Fig. 9), and some possible intermediate structures were deduced by referring to the GC-MS library with the molecular ion and mass fragmentation pattern (Table. S1). Firstly, *·*O_2_^*−*^ and ·OH were generated at the surface of the catalyst during the redox process and thus RhB was attacked to decolorize the dye solutions and was further degraded by active species. The primary intermediates were low-molecule-weight benzoic acid and their derivatives after oxidized and opened degradation. Eventually, the benzene rings were continuously attacked with the progress of photocatalytic reaction, which would be degraded to some acids and decomposed into CO_2_ and H_2_O.

**Figure 10:**
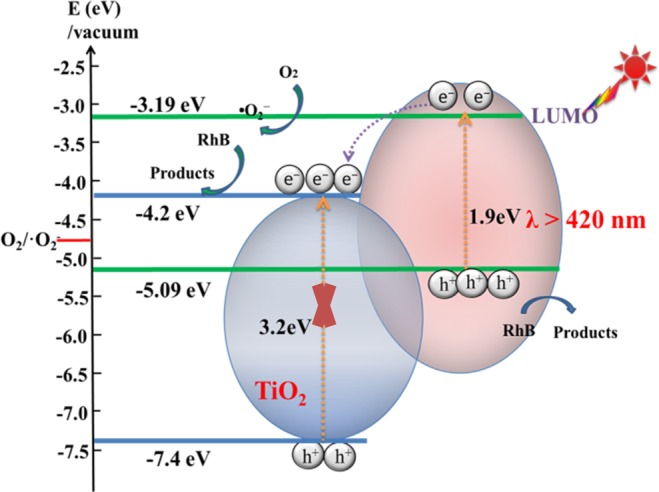
Schematic illustration of heterojunction formed between the polymer and TiO_2_. (This image is created by WPS Office. https://www.kdocs.cn/welcome#home).

As a result of the above experiments, the possible photocatalytic reaction mechanism of RhB based on PTET-T/TiO_2_ and PTET-T-COOH/TiO_2_ heterostructure was proposed. The optical band gap energy of PTET-T and PTET-T-COOH were similar and were determined to be 1.9 eV according to the absorption edge of DRS spectra. In addition, the results of CV (Figure S3) showed the highest occupied molecular orbital (HOMO) of PTET-T and PTET-T-COOH were −5.19 eV and −5.09 eV, respectively. Meanwhile, the lowest unoccupied molecular orbital (LUMO) of were calculated at −3.29 eV and −3.19 eV (vs. Vacuum) respectively, which indicated that the electrons on the LUMO of conjugated polymers could reduce O_2_ molecules to generate ·O_2_^−^. It is well known that the valence band (VB) and conduction band (CB) of commercial TiO_2_ at −7.4 eV and −4.2 eV^50^ (vs. Vacuum), respectively. As shown in Fig. 10, the conjugated polymers in the well-aligned straddling heterojunction structures of conjugated polymers/TiO_2_ were firstly excited under the visible light irradiation, which further produced the electron-hole pair and transferred to the LUMO and HOMO corresponding to electron and hole, respectively. Then the electrons on LUMO of conjugated polymers would transfer to CB of TiO_2_ participating in the oxidation reactions. At the same time, the holes on HOMO of conjugated polymers could directly oxidize organic pollutants. However, the LUMO of PTET-T-COOH (−3.19 eV vs. NHE) is not positive enough to oxidize the water or surface hydroxyl group to generate ·OH. The active radical capture experiments show that the ·OH radicals have an influence on the photocatalytic performance which is corresponding with the ESR results. Thence, there is no doubt that the strong signals of DMPO-·OH may arise from further conversion of ·O_2_^−^^51,52^. The specific reaction process was as follows:

$$\text{C}1/\text{C}2+\text{hv}\to \text{C}1/\text{C}2({e}^{-})+\text{C}1/\text{C}2({{\rm{h}}}^{+})$$

$${\text{h}}^{+}+\text{RhB}\to \to \text{Degradation}\,\text{product}$$

$${\text{TiO}}_{2}+{\text{e}}^{-}+{\text{O}}_{2}\to {\text{TiO}}_{2}({\text{e}}^{-})+\cdot {{\text{O}}_{2}}^{\mbox{--}}$$

$$\cdot {{\text{O}}_{2}}^{\mbox{--}}+\text{RhB}\to \to \text{Degradation}\,\text{product}$$

$$\cdot {{\text{O}}_{2}}^{\mbox{--}}+{\text{H}}_{2}\text{O}\to {\text{H}}_{2}{\text{O}}_{2}\to \cdot \text{OH}$$

$$\cdot \text{OH}+\text{RhB}\to \to \text{Degradation}\,\text{product}$$

## Conclusions

In conclusion, the novel conjugated polymers PTET-T and PTET-T-COOH were synthesized and the PTET-T/TiO_2_ and PTET-T-COOH/TiO_2_ heterojunctions in different proportions of the complexes were further successfully fabricated. The “lock-in effect” in PTET-T-COOH/TiO_2_ formed in the interfacial interaction between carboxyl groups and hydroxyl groups was testified, which greatly improves the electron-hole separation efficiency and interface charge transfer ability. As a result, the 15% PTET-T-COOH/TiO_2_ illustrated the highest photocatalytic degradation ability with removing almostly entire RhB in 80 min, and its kinetic rate constant was 41.7 times of that of TiO_2_. Moreover, 15% PTET-T-COOH/TiO_2_ also showed effective photocatalytic activity for MB and TC. The h^+^, ·OH, and ·O_2_^−^ active species have been proved to play the critical roles during the photodegradation. This work not only provided a strategy for designing and synthesizing novel conjugate polymers, but also presented promising candidates for photocatalytic degradation field.

## Experimental section

### Materials

Titanium dioxide (TiO_2_, anatase), titanium tetrachloride (TiCl_4_), thiophenethiophene-3-carboxylic acid, N-bromosuccinimide (NBS) thiophene-2-carbaldehyde, trimethyl chlorotin (C_3_H_9_ClSn) and tetrakis (triphenylphosphine)palladium Pd (PPh_3_)_4_ were purchased from Sigma-Aldrich Chemical Co. All the reagents were analytical reagent and used as is without additional purification.

### Synthesis

**Scheme 1:**
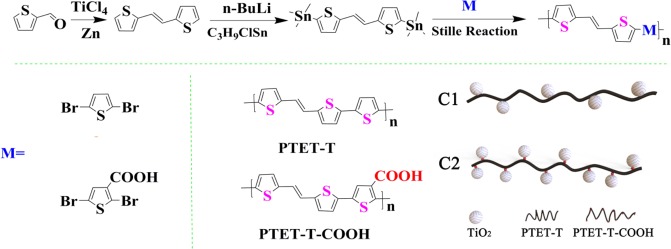
Synthesis of the conjugated polymer PTET-T, PTET-T-COOH and Schematic diagram of heterojunction C1 and C2. (This image is created by InDrawforWeb. http://indrawforweb.integle.com/).

The conjugated polymer PTET-T and PTET-T-COOH were easily obtained in three steps, according to Scheme 1. Firstly, (E)-1,2-di(thiophen-2-yl)ethane 1^53^, (E)-1,2-bis(5-(trimethylstannyl)thiophen-2-yl)ethane 2^54^, 2,5-dibromothiophene 3^55^ and 2,5-dibromothiophene-3-carboxylic acid 4^56^ had been synthesized according to the reported procedure, respectively. The desired polymer PTET-T and PTET-T-COOH were yielded through Stille coupling polymerization of (E)-1, 2-bis(5-(trimethylstannyl)thiophen-2-yl)ethane 2 with 2,5-dibromothiophene 3 and 2,5-dibromothiophene-3-carboxylic acid 4 in anhydrous toluene. Then, a certain amount of Pd(PPh_3_)_4_ catalyst was added to reaction and heated at 110 °C for 3d. The specific synthesis processes were given in supporting materials.

The heterojunctions (C1, C2) were prepared as the under process: a given mass of polymer particles and 20 mL of THF were put into round-bottom flask for 60 min at room temperature and then the mixed solution was dropwise add into 100 mL of TiO_2_ alcohol solution. The suspension was fully stirred for 120 min at dark to sure that the composites were completely dispersed. Whereafter, the products were concentrated by rotary evaporation and dried at 40 °C under vacuum oven for 72 h. The heterojunctions were successfully synthesis. The heterojunctions with different polymer ratios from 1 wt% to 50 wt% were prepared according to the above method. The color of the final products varied from light-brownish red to deep-brownish red as different polymer amounts were loaded on TiO_2_.

### Instrumentation

The morphology and microstructure were characterized by a field emission scanning electron microscopy (FESEM, JEOL 7800 F) and a transmission electron microscopy (TEM, FEI Tenai G2 F20). The chemical structure was confirmed on a solid state magic angle spinning (MAS) ^13^C NMR spectrometer (Bruker Avance 400 MHz) and a Fourier transform infrared (FT-IR) spectrometer (Nicolet Is50). The product constituent analyses were characterized by X-ray powder diffraction (XRD) with Cu-K_α_ radiation at 40 kV and 200 mA (λ = 1.5406 Å). X-ray photoelectron spectra (XPS) were performed with an ES- CALAB250XI electron spectrometer (VG Scientific, America) using 300 W Al K_α_ radiation. UV-vis diffuse reflection spectra (DRS) of the samples were analyzed with BaSO_4_ as the background by using a scan UV–vis spectrometer (Perkin-Elmer Lambda 900). Photoluminescence (PL) (time-resolved) spectra were recorded using a continuous 365 W Xe lamp/Xe flash tube-equipped spectrofluorometer (Horiba JobinYvon Fluorolog-3), respectively. The photoelectrochemical measurements were performed by using an electrochemical workstation (PGSTAT302/FRA2/ECN/ECD/BIPOT, Autolab). The electron spin resonance (ESR) was conducted using a (JEOL) JESFA200 spectrometer. The electrochemical cyclic voltammetry (CV), photocurrent and EIS were performed on a CHI 660 C electrochemical workstation (Chenhua Instruments Co. Shanghai china).

### Photocatalytic activity measurement

The photocatalytic activities of the photocatalysts under visible-light irradiation, which is CEL-HXUV300 300 W Xe lamp with a UV cutoff to filtrer out the irradiation with wavelength below 420 nm, were evaluated by the target molecules of RhB, methylene blue (MB) and tetracycline (TC). The average visible light intensity was 100 mW cm^−2^. For the measurement, 20 mg of photocatalyst and the 100 mL of 10 mg/L organic pollutants solution were dispersed in a reaction vessel with a water cooling system. In order to make sure that the adsorption-desorption equilibrium between the photocatalyst and the organic pollutants system, the suspension was continuously stirred in darkness for 60 min. 4 mL of sample was carried out every 20 min and centrifuged the catalyst at 4000 r min^−1^ for 5 min. The supernatant liquid was analyzed by recording the maximum band of organic pollutants with a UV-vis spectrophotometer (UV-5800PC, Shanghai Metash Instruments Co., Ltd). The photodegradation efficiency = C/C_0_ × 100%, where C_0_ and C correspond to the concentration of pollutants at the initial and real time^57^. The recycled photocatalyst was washed with ethanol and deionized water for several times, then dried in a vacuum at 40 °C for 24 h. Besides, the degradation of possible products was detected by Gas chromatography and mass spectrometry (GC-MS). Total organic carbons were measured on a TOC analyzer. (AnalytikJena AG, Germany).

## Supplementary information

Supplementary Information.
