# Supplementary material for: A novel Strategy of Lock-in Effect between Conjugated Polymer and TiO2 towards Dramatic Enhancement of Photocatalytic Activity under Visible Light
Source: Sci Rep. 2020 Apr 16;10:6513. doi: 10.1038/s41598-020-63623-2 (PMC7162871; doi:10.1038/s41598-020-63623-2)
Supplement: Supplementary file 1 — Supplementary Information. [file 41598_2020_63623_MOESM1_ESM.docx]

**Supplementary Material**

A novel Strategy of Lock-in Effect between Conjugated Polymer and TiO_2_ towards Dramatic Enhancement of Photocatalytic Activity under Visible Light

**Linlin Liu**^1, 2, 3^**, Wei Jiang**^2, 3^**, Xingyue Song**^4^**, Qian Duan**^*1^, **and Enwei Zhu**^**2, 3^

^1^ School of Materials Science and Engineering, Changchun University of Science and Technology, Changchun 130022, P. R. China

^2^ Key Laboratory of Functional Materials Physics and Chemistry of the Ministry of Education, Jilin Normal University, Changchun 130103, P. R. China

^3^ Key Laboratory of Preparation and Application of Environmental Friendly Materials, Jilin Normal University, Ministry of Education, Changchun 130103, P. R. China

^4^ School of Environmental Science and Engineering, Jilin Normal University, Siping 136000, P. R. China

*Corresponding author, Qian Duan, Email, duanqian88@hotmail.com.

**Corresponding author, Enwei Zhu, Email, [zhuenwei05@126.com](mailto:zhuenwei05@126.com).

1. Detailed experimental section

**(E)-1, 2-di(thiophen-2-yl)ethane 1^[1]^**

Thiophene-2-carbaldehyde (5 g, 44.6 mmol) and freshly distilled THF (50 mL) were added in a schlenk tube at -18°C and then titanium tetrachloride (1 mol/L in THF, 18 mL) was added dropwise in the tube. The solution was stirred for one hour at the same temperature and Zn (21 g, 0.32mol) in batches was added and refluxed for four hours. Quenched with Na_2_CO_3_, the pH value of the reaction mixture was buffered close to 6-8, and extracted with CH_2_Cl_2_ (3 × 20 mL). The combined organic layers were dried over anhydrous MgSO_4_. After solvent removal, the residue was subjected to chromatography over silicagel using ethyl acetate/petroleum ether (1/20, v/v) as eluent. The title compound was obtained as a yellow solid (4 g, 80%).

**(E)-1, 2-bis(5-(triMethylstannyl)thiophen-2-yl)ethane 2^[2]^**

To a solution of **1** (2 g, 4 mmol) in anhydrous THF (50 mL) at -78 °C was added n-BuLi (2.4 mL, 5.77 mmol, 2.4 M in hexane) under N_2_. After two hours of being stirred at -78 °C, the reaction mixture was warmed to room temperature and stirred 30 min. Then the mixture was cooled to -78 °C again. Trimethyltin chloride (2.3 g, 12 mmol) was dissolved in THF (5 mL) and added into the tube. The resulting mixture was stirred for two hours before being warmed to room temperature and allowed to stir overnight. Quenched with H_2_O, the reaction mixture was extracted with CH_2_Cl_2_ and dried over MgSO_4_. After solvent removal, the residue was purified by recrystallization from ethanol. The pure product was obtained as yellow needlelike solid (1.54 g, 77%).

**2,5-dibromothiophene 3^[3]^**

The [thiophene](app:ds:thiophene) (5 g, 0.06mmol) and acetic acid (20 mL) were dissolved in CHCl_3_ (100 mL), then slowly dropwise added to a solution of *N*-bromosuccinimide (NBS, 22 mg, 0.125 mmol) dissolved in CHCl_3_ (50 mL) at 0 °C. The mixture was stirred overnight at room temperature. Then the mixture was poured into a saturated sodium bisulfite solution (50 mL), and the organic layer was washed with water. The product was purified using column chromatography (silica gel, petroleum ether) to afford the title compound as a dark red liquid (4.51 g, 90.2%).

**2,5-dibromothiophene-3-carboxylic acid 4^[4]^**

2,5-dibromothiophene-3-carboxylic acid **4** was prepared by the literature method. Thiophene-3-carboxylic acid (1.91 g, 15 mmol) and *N*-bromosuccinimide (NBS) (5.40 g, 30.29 mmol) were dissolved in anhydrous THF (30 mL). The mixture was photophobic and then stirred at 50°C for 28 h under N_2_ before cooled to room temperature. The mixture was purified in water. The resulting precipitate was collected by filtration and washed with brine, dried over anhydrous MgSO_4_, and then filtered to afford pure target product (1.69 g, 88.5%).

**Conjugated Polymer PTET-T and PTET-T-COOH**

The mixture solution of (E)-1,2-bis(5-(trimethylstannyl)thiophen-2-yl)ethane **2** (2.0 g, 3.86 mmol) and 2,5-dibromothiophene **3** (934 mg, 3.86 mmol) or 2,5-dibromothiophene-3-carboxylic acid **4** (1.1 g, 3.86 mmol) in anhydrous anaerobic toluene (200 ml) was purged with nitrogen for 30 min to remove O_2_. After the addition of Pd(PPh_3_)_4_ (3 mol % equiv, 60 mg), the reaction mixture was stirred at 110 °C for 72 h. The polymer was precipitated from a methanol solution and extracted with hexane and methanol for 24h to give a red solid (1.12 g, 56%).

2. Additional Experimental Results





**Figure S1.** Adsorption experiments of RhB in the presence of PTET-T, pure TiO_2_, C1, and C2 composites.


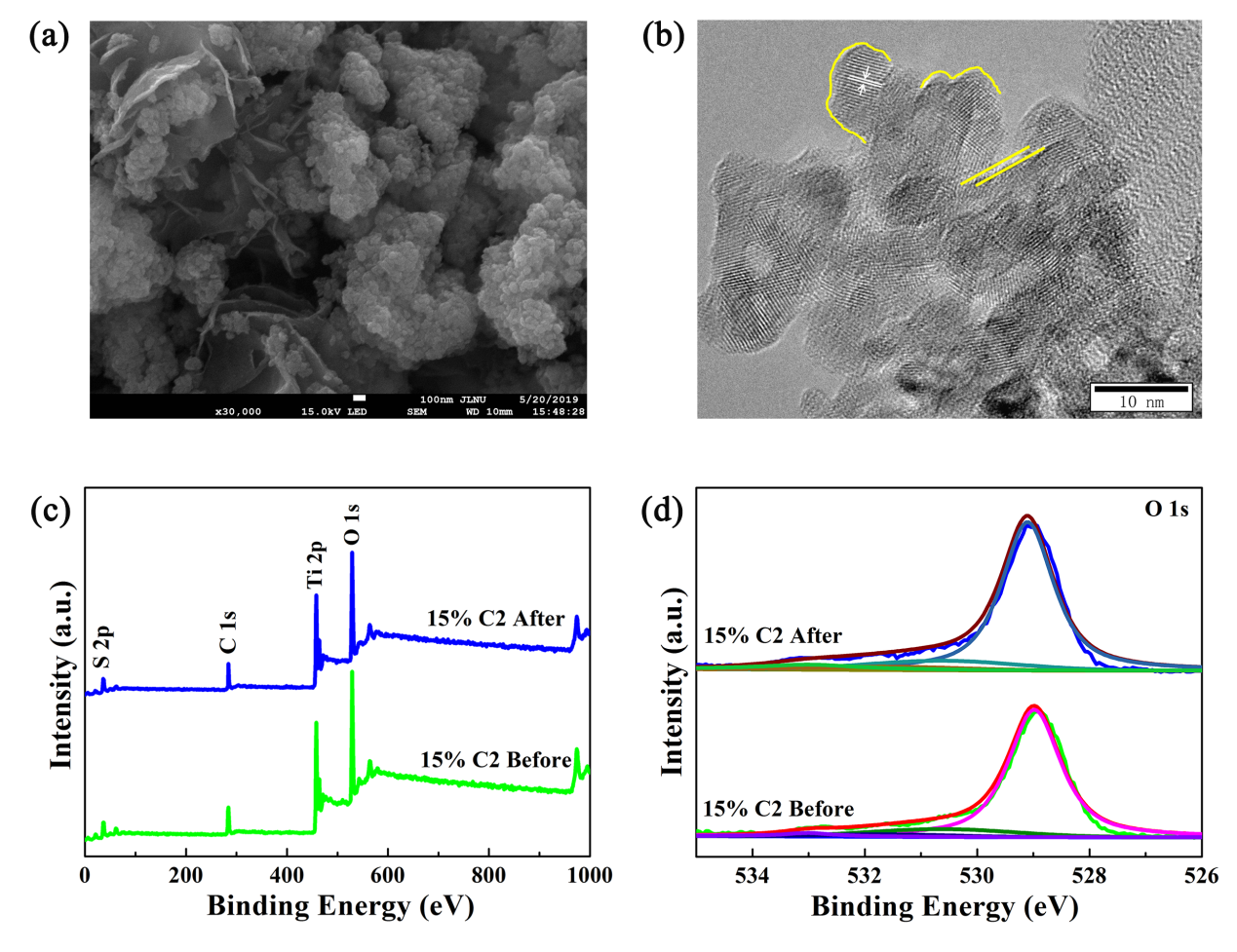


**Figure S2.** (a) SEM, (b) TEM, and XPS spectra (c) survey spectrum, (d) O 1s of 15% C2 before and after four times photocatalytic degradation of RhB





**Figure S3.** Cyclic voltammograms of PTET-T and PTET-T-COOH.

**Table S1** Retention times and the identified products corresponding to **Figure 9**.

| Time | Molecular Formula | m/z | Name | Structural Formula |
| --- | --- | --- | --- | --- |
| 18.94 | C_7_H_6_O_4_ | 154 | 2,4,6-Trihydroxybenzaldehyde |  |
| 18.94 | C_7_H_6_O_4_ | 154 | 2,3,4-Trihydroxybenzaldehyde |  |
| 18.94 | C_7_H_6_O_2_ | 122 | Benzoic acid |  |
| 18.94 | C_7_H_6_O_4_ | 154 | 2,5-Dihydroxybenzoic acid |  |
| 18.94 | C_7_H_6_O_3_ | 138 | 3-Hydroxybenzoic acid |  |
| 18.94 | C_7_H_6_O_4_ | 154 | 2,6-Dihydroxybenzoic acid |  |
| 18.94 | C_8_H_8_O_3_ | 152 | 3-Methylsalicylic acid |  |
| 3.239 | C_2_H_5_NO | 59 | Acetamide |  |
| 3.239 | C_4_H_9_NO | 87 | Butanamide |  |
| 3.239 | C_4_H_9_NO | 87 | Ethanimidic acid, ethyl ester |  |
| 3．239 | CH_2_O_2_ | 46 | Formic acid |  |
|  | | | | |

References

1. Nguyen, T. P. Polymer-based nanocomposites for organic optoelectronic devices. A review. *Surf. Coat. Technol.* **206**, 742-752 (2011).

2. Neculqueo, G., Fuentes, V. R., López, A., Matute, R., Vásquez, S. O. Electronic properties of thienylene vinylene oligomers: synthesis and theoretical study. *Struct. Chem.* **23**, 1751-1760 (2012).

3. Fan, Q.P., Li, M., Yang, P.G., Liu, Y., Xiao, M.J. *et al.* Acceptor-donor-acceptor small molecules containing benzo[1,2- b :4,5- b ']dithiophene and rhodanine units for solution processed organic solar cells. *Dyes Pigm.* **116**, 13-19 (2015).

4. Li, Y.S., Liu, M.X., Chen, L. Polyoxometalate built-in conjugated microporous polymers for visible-light heterogeneous photocatalysis. *J Mater Chem A.* **5***,* 13757-13762 (2017).
